# Application of Machine Learning in the Diagnosis of Early Gastric Cancer Using the Kyoto Classification Score and Clinical Features Collected from Medical Consultations

**DOI:** 10.3390/bioengineering11100973

**Published:** 2024-09-27

**Authors:** Xue Sun, Liping Zhang, Qingfeng Luo, Yan Zhou, Jun Du, Dongmei Fu, Ziyu Wang, Yi Lei, Qing Wang, Li Zhao

**Affiliations:** 1 Department of General Practice, Beijing Hospital, National Center of Gerontology, Institute of Geriatric Medicine, Chinese Academy of Medical Sciences, Beijing 100730, China; sunxue4682@bjhmoh.cn (X.S.); zhouyan3364@bjhmoh.cn (Y.Z.); 2 Pharmacovigilance Research Center for Information Technology and Data Science, Cross-Strait Tsinghua Research Institute, Xiamen 361015, China; lipingzhang94@outlook.com; 3 Department of Gastroenterology, Beijing Hospital, National Center of Gerontology, Institute of Geriatric Medicine, Chinese Academy of Medical Sciences, Beijing 100730, China; luoqingfeng2554@bjhmoh.cn (Q.L.); fudongmei@163.com (D.F.); 4 Department of Pathology, Beijing Hospital, National Center of Gerontology, Institute of Geriatric Medicine, Chinese Academy of Medical Sciences, Beijing 100730, China; dujun3255@bjhmoh.cn; 5 Digestive Endoscopy Center, Beijing Majiapu Community Health Service Center, Beijing 100068, China; wangziyu@163.com

**Keywords:** early gastric cancer, Kyoto classification, machine learning, endoscopy, KANs

## Abstract

The early detection accuracy of early gastric cancer (EGC) determines the choice of the optimal treatment strategy and the related medical expenses. We aimed to develop a simple, affordable, and time-saving diagnostic model using six machine learning (ML) algorithms for the diagnosis of EGC. It is based on the endoscopy-based Kyoto classification score obtained after the completion of endoscopy and other clinical features obtained after medical consultation. We retrospectively evaluated 1999 patients who underwent gastrointestinal endoscopy at the China Beijing Hospital. Of these, 203 subjects were diagnosed with EGC. The data were randomly divided into training and test sets (ratio 4:1). We constructed six ML models, and the developed models were evaluated on the testing set. This procedure was repeated five times. The Kolmogorov–Arnold Networks (KANs) model achieved the best performance (mean AUC value: 0.76; mean balanced accuracy: 70.96%; mean precision: 58.91%; mean recall: 70.96%; mean false positive rate: 26.11%; mean false negative rate: 31.96%; and mean F1 score value: 58.46). The endoscopy-based Kyoto classification score was the most important feature with the highest feature importance score. The results suggest that the KAN model, the optimal ML model in this study, has the potential to identify EGC patients, which may result in a reduction in both the time cost and medical expenses in clinical practice.

## 1. Introduction

Gastric cancer (GC) is one of the most common cancers worldwide. Despite a decline in the incidence of GC over recent decades, the incidence rate of GC in China accounts for 44% of the world’s gastric cancer incidence rate [1]. In China, gastric cancer was the third leading cause of cancer-related mortality, with 260,400 deaths recorded in 2022 [2]. GC is a multi-factorial disease with environmental and genetic factors contributing to its etiology. Some of these risk factors, such as age, sex, and family history of gastric cancer, are not modifiable. Conversely, other factors, such as *Helicobacter pylori* (HP) infection, nutrition improvement, body mass index (BMI), smoking and alcohol consumption, and physical activity, can be modified [3,4]. Gastric cancer can be classified into two types based on staging: early stage and advanced stage. Patients with EGC can be cured with timely treatment. Nevertheless, the five-year survival rate is poor for those diagnosed at an advanced stage, even after surgery [5].

Clinically, gastrointestinal endoscopy is the primary and preferred means of screening GC [6]. Unfortunately, due to hidden symptoms of EGC, some patients with EGC do not actively choose to undergo endoscopy until the symptoms become severe, which leads to most of the GC patients being diagnosed at an advanced stage [7]. Therefore, the early detection for EGC is vital, especially to those with high risk factors, and would reduce the costs associated with follow-up care and prevent the progression of gastric cancer at its earliest possible stage.

In recent years, gastrointestinal endoscopy is gradually popularized in primary hospitals. Nevertheless, due to the considerable number of patients and the relatively poor quality of medical conditions in primary hospitals, their endoscopists were forced to make definitive diagnosis based solely on the results of endoscopy that performed with their eyes. As it is possible that the cancerous areas may be missed detected or incorrectly identified, the rate of missed diagnosis of EGC is not optimistic [8,9,10]. In contrast, a definitive diagnosis of EGC in a municipal hospital is usually based on a combination of gastrointestinal endoscopy and the results of three kinds of gastric functional tests (i.e., the PG I test, PG II test, and G-17 test) or a single gastric functional test [11]. However, the additional cost of gastric functional tests made them unaffordable for a proportion of patients in China, which may have further discouraged proactive patient attendance.

In the field of gastroenterology, several prediction models for gastric cancer that have been developed by ML methods, which process tabular datasets instead of endoscopic images, have been reported in previous studies [12,13,14]. ML techniques are an emerging tool for the development of both predictive models and data analysis models. It can implicitly extract useful information from raw data, even uncovering patterns [15]. However, the previously described EGC prediction models were all constructed using traditional ML algorithms that have achieved good performance on tabular datasets, such as the random forest (RF) algorithm [16,17], multi-layer perceptron (MLP) algorithm [17,18], extra trees (ET) algorithm [19], support vector machine algorithm [17,20], gradient-boosted trees algorithm [16,17,20], and logistic regression (LR) algorithm [16,18], rather than KAN algorithms. KANs represent a new class of neural network architectures that draw inspiration from the work of Andrey Kolmogorov and Vladimir Arnold [21,22]. By representing functions as the sum of these learnable functions, a KAN can accurately represent continuous functions while maintaining the interpretability of the underlying model [23]. KANs were introduced specifically as an alternative to MLPs [23]. In the study conducted by Eleonora Poeta et al. [23], KANs exhibited superior or comparable accuracy and F1 scores in datasets with numerous samples compared to MLPs, indicating that it can robustly handle complex tabular data. In comparison to complex models with limited interpretability, selected ML models that constructed traditional ML algorithms have clear interpretability and achieved good performance in addressing classification problems with tabular datasets [24,25,26]. Consequently, five traditional ML algorithms (i.e., ET, RF, LR, Ada Boost Classifier (Ada Boost), and Radial Basis Function Kernel Support Vector Machine Classifier (RBF-SVM)) were selected for EGC prediction in our study, in comparison to the KAN algorithm.

Concurrently, with respect to independent variables involved in the development of EGC prediction model, few studies have considered the endoscopy-based Kyoto classification score, also called Kyoto Classification of Gastritis, as a significant risk factor for constructing ML models for EGC prediction [4]. The endoscopy-based Kyoto classification score was calculated following the completion of endoscopy, which was first advocated at the Japan Gastroenterological Endoscopy Society in 2013 and has since become one of the most commonly used gastritis classification systems worldwide [27]. A higher Kyoto classification score indicates higher risk of current HP infection and gastric cancer [28]. The majority of previous studies have considered patients’ personal information, endoscopic features (i.e., atrophy, intestinal metaplasia, enlarged fold, nodularity, and diffuse redness), and the results of routine blood, biochemical, and tumor marker tests as independent variables in the development of ML models for the diagnosis of EGC [13,16,17,20]. In comparison to a number of endoscopic characteristics, the endoscopic Kyoto classification score of gastritis gives us a unified way to describe gastritis of different individuals [4], which makes it suitable as an important variable to improve the performance of EGC prediction model. For example, the work of Lin J et al. demonstrated that a predictive nomogram model, constructed based on the endoscopic Kyoto classification scores, age, sex, PG I/II ratio, HP antibody, and four endoscopic features, was proven to be of high predictive value for GC [4]. Nevertheless, their method of predicting gastric cancer requires the information on the PG I/II ratio, which resulted in increased medical expenses and a time delay in the final diagnosis.

Thus, we hypothesize that ML-based prediction models of EGC that take into account a minimum number of effective variables, including the endoscopy-based Kyoto classification score and other clinical features collected after a simple medical consultation, can be a simple and affordable method for the prediction of EGC in hospitals. The aforementioned clinical features include age, gender, BMI, family history of gastric cancer, history of HP infection and HP eradication, and smoking and alcohol consumption. Six ML models were constructed for the purpose of predicting EGC. The aforementioned ML methods included five traditional ML methods and a novel ML method, namely, KANs.

The objective of the present study was to evaluate and sought out the optimal ML model using the endoscopy-based Kyoto classification score and multiple clinical features collected from medical consultations in order to develop a practical and low-cost diagnostic method for EGC.

The three main contributions of our work are as follows: (1) Investigating suitable machine learning approaches for the diagnosis of EGC among six ML models (i.e., ET, RF, LR, Ada Boost, and RBF-SVM, and the KAN model) using the Kyoto classification score and other clinical features collected from medical consultations. (2) Finding the most important feature with the highest feature importance score for predicting EGC cases. (3) The KAN model, which was constructed using a novel ML method (i.e., KANs) for EGC prediction, was proposed as the optimal ML model for implementation in clinical practice following gastrointestinal endoscopy and routine consultations.

## 2. Materials and Methods

### 2.1. Patients and Inclusion and Exclusion Criteria

This is a retrospective, single-center study that received approval from the Ethics Committee of Beijing Hospital, Chinese Academy of Medical Sciences (approved no. 2020BJYYEC-061-08) and was registered at the Chinese Clinical Trial Registry (ChiCTR2000032812). Data were collected from May 2020 to May 2024, involving 2042 patients aged 23–91 years with suspected chronic gastritis. All participants provided written informed consent to cooperate with routine diagnostic gastroscopy. Inclusion criteria are as follows: (1) The patient underwent gastroscopy at our hospital’s Digestive Endoscopy Center. (2) The patient signed an informed consent form and could cooperate with the gastroscopy, including white-light gastroscopy and LCI mode. (3) Pathologically confirmed as gastric cancer or no gastric cancer through biopsy or endoscopic submucosal dissection. (4) White-light gastroscopy and LCI images of the suspected gastric cancer area were clear. The exclusion criteria are as follows: (1) Inability to tolerate conventional endoscopy. (2) Already diagnosed with middle or advanced stage gastric cancer. (3) Pathologically confirmed as fundamental gastric cancer, gastrinoma, gastric adenocarcinoma of fundic gland, or signet ring cell carcinoma. (4) Missing data of one of variables in this study.

We numbered the included patients firstly and collected basic information, including gender, age, height, weight, HP infection and HP eradication history, and family history of gastric cancer; lifestyle information was also collected, including alcohol consumption and smoking consumption. The status of *Helicobacter pylori* infection is determined through the collection of medical history. For example, if a patient had previously been infected but was successfully eradicated, this information was recorded. However, during subsequent endoscopic examinations, if the rapid urease test was positive, or if *Helicobacter pylori* was detected in the gastric mucosal biopsy, the information would be revised accordingly. The BMI of the patient was calculated using the patient’s weight and height values and was subsequently graded. In this study, the endoscopy-based Kyoto classification score of each case was evaluated by at least two experienced endoscopists, with the score ranging from 0 to 8. If the two endoscopists had different opinions, then they sought the advice of a third senior endoscopist. The Kyoto classification score is defined as the sum of five endoscopic findings, including atrophy score, intestinal metaplasia score, diffuse redness score, nodularity score, and enlarged folds score [29,30].

**Figure 1:**
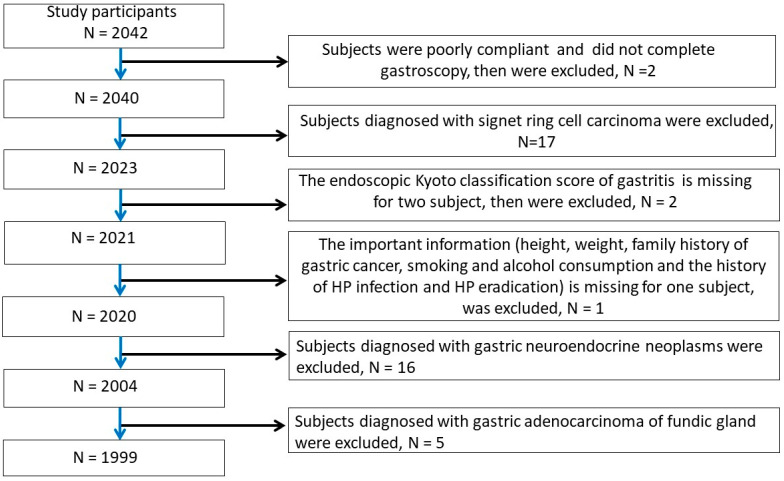
The process of inclusion and elimination.

**Figure 2:**
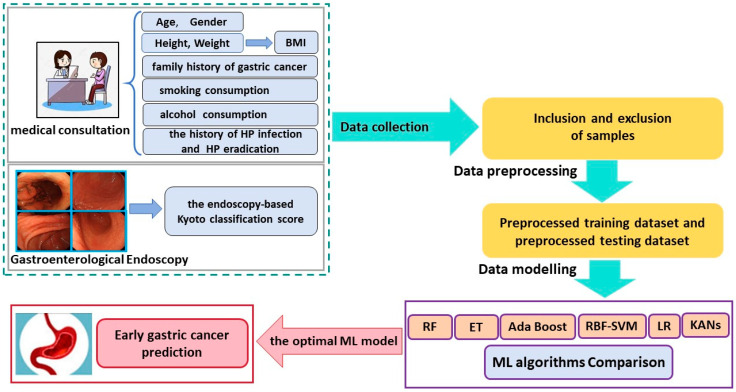
The workflow of current research.

In this study, 43 patients were excluded based on according to the aforementioned exclusion criteria. A total of 1999 subjects were enrolled for further study. The details of sample inclusion and exclusion are shown in Figure 1. The workflow of the current research is shown in Figure 2.

### 2.2. Raw Data Preprocessing

Each sample in the raw dataset comprised eight features and one label column. These eight features are age, gender, BMI, family history of gastric cancer, history of HP infection and HP eradication, smoking consumption, alcohol consumption, and the endoscopy-based Kyoto classification score. All of these variables are categorical features. The label values were encoded as 1 for the positive class and 0 for the negative class. The samples with the positive class label and the negative class label represent EGC cases (*n* = 203) and non-EGC cases (*n* = 1796), respectively, which indicates that the two classes of data were lightly imbalanced.

Firstly, no imputation was performed on categorical features, as these features had no missing values after the process of data exclusion. One-hot encoding was applied to the categorical features. Secondly, the datasets were randomly divided into a training set (1599 samples) and a testing set (400 samples) with an 8:2 ratio. Thirdly, in order to alleviate the problem of the biased results towards the majority due to the two classes of data being imbalanced, the “WeightedRandomSampler” function, a type of sampler from the Pytorch library (specifically, from torch.utils.data.sampler), was employed to calculate the probabilities of the sample with label 1 and label 0.

In our study, the output of the “WeightedRandomSampler” function is a sequence of weights and the length of which is equal to that of the training set. Subsequently, the minority samples were resampled from the training set with the given weights sequence and drawn with replacement through ten iterations. In each iteration, 10% of samples of training set were resampled. Following the resampling procedure, the ratio of samples with a positive class label to those with a negative class label in the preprocessed training set is approximately equal to 1:1. Table 1 lists the number of positive samples and negative samples in the training and testing sets, respectively, following data preprocessing. The above resampling process was applied to the training set only and left the testing set intact in order to avoid the inflation of performance measures.

### 2.3. Machine Learning Methods and Evaluation Metrics for Classification

Instead of complex models with limited interpretability, six ML techniques were employed for data modelling and evaluated for EGC binary classification performance using actual clinical data during cross-validation [31], as reported in previous studies [17,18,20,32,33]. The ML methods in this study included LR [33], RBF-SVM [34,35], ET [36], Ada Boost [37], RF [38], and KAN [39] algorithms.

LR is a widely used multi-variable method for binary classification [40]. Logistic regression models are employed to examine the effects of multiple predictor variables on the outcome and normally the outcome is binary, such as the presence or absence of disease [41].

Support vector machine is an optimal margin-based classification technique that transforms the original data into a higher-dimensional space using nonlinear mapping. In a higher-dimensional space, the support vector machine employs support vectors to find an optimal hyperplane that separates the original data points according to their annotation profiles [42]. For solving the binary classification task efficiently, the kernel function of SVM was the radial basis function (RBF) in this study.

Ada Boost is an ensemble method that improves the performance of a base machine learning algorithm [43]. After adjusting the sample weights and the weights of weak learners, the output of the base learning algorithms is combined into a weighted sum that represents the final output of the boosted classifier. Subsequent base learners are trained in favor of those samples that were misclassified by previous classifiers [37].

A random forest is a classifier consisting of a set of tree-structured classifiers where each tree depends on the values of a random vector sampled independently and with the same distribution for all trees in the forest [38]. In essence, random forest generates a multitude of decision trees, which are used to form a “forest”. The output is then determined by a voting process conducted on the multiple trees that comprise the forest. However, its performance is strictly related to a number of hyperparameters, such as the number of trees in the forest and the pruning strategies, as well as the max depth value.

ET is an extremely randomized tree-growing algorithm that combines the attribute randomization of random subspace with a totally random selection of the cut-point [36]. This method depends on the number of trees M, one main parameter K, and a secondary parameter n_min_. The main parameter K controls the strength of the attribute randomization and the secondary parameter n_min_ controls the degree of smoothing [36].

As proposed by Liu et al. [39], KANs are promising alternatives to MLPs and represent a novel class of neural network architectures. In traditional neural networks, the nodes of traditional neural networks employed fixed nonlinear activation functions.

In contrast, each edge in a KAN is characterized by a learnable activation function., i.e., the parameter of each weight in the edges of KANs is replaced with a learnable 1D function that is typically parameterized as a spline [23]. By representing functions as a sum of these learnable functions, KANs can represent continuous functions accurately while maintaining the interpretability of the underlying model [23]. This potentially significantly enhances accuracy and interpretability in function approximation tasks [39].

A number of standard metrics were employed for the performance evaluation of each ML algorithm in our study. These metrics included the area under the receiver operating characteristic curve (AUC), balanced accuracy (BA), F1 score, precision, recall, false positive rate (FPR), and false negative rate (FNR). For calculating the evaluation metrics of datasets with binary targets in this study, we report the macro (i.e., unweighted) average [23]. Balanced accuracy represents a corrected measure of accuracy that is used for the purpose of comparing datasets with imbalances in sample size. The value of AUC is the area under the receiver operating characteristic (ROC) curve, which was employed to evaluate and seek out the optimal ML model. Higher AUC values indicate better classification performance of the model [44]. The predefined probability threshold was set to 0.5. Python 3.6 and the open-source Python automated machine learning library, PyCaret 2.3.10, were used for data modeling of traditional ML methods, model evaluation of traditional ML methods, and feature analysis. The Pytorch 1.10.2 library was used for data preprocessing. The scikit-learn library was used for the calculation of evaluation metrics.

### 2.4. The Procedure of Data Modelling of Traditional ML Methods

**Figure 3:**
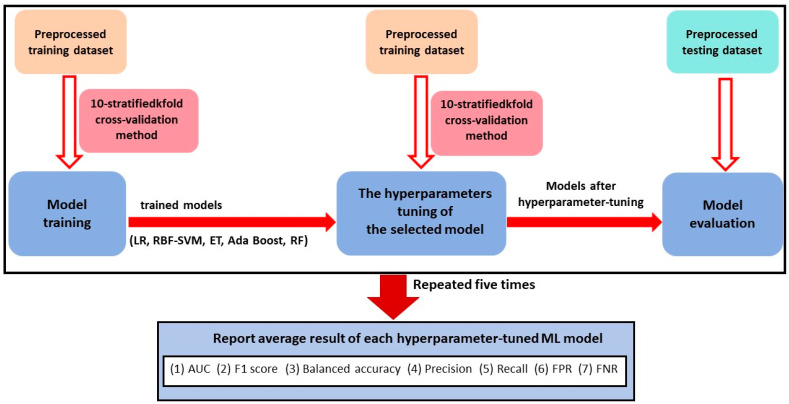
The block diagram of the process of data modeling of five traditional ML models.

The data modelling procedure of the five traditional ML models, as illustrated in Figure 3, was divided into three stages: model training, hyperparameter tuning of the selected model, and model evaluation. The traditional ML models included in this study were LR, RBF-SVM, ET, Ada Boost, and RF. The preprocessed training datasets were used for model training and optimization using 10-stratified k-fold cross-validation. A random grid search was employed for hyperparameter tuning, with the highest AUC value serving as the target for model hyperparameter tuning.

Then, the optimal hyperparameters and the preprocessed training datasets were employed to train and obtain each hyperparameter-tuned ML model. Seven metrics were derived from each hyperparameter-tuned ML model and employed for the performance evaluation of each ML algorithm on the testing sets in each data modeling process for each ML model. A comparison of the results of the training model with those of the hyperparameter-tuned model can be used to assess the appropriateness of the selected hyperparameters. Furthermore, a comparison of the results of the tuned model on the training dataset with those of the tuned model on the test dataset is required, in order to evaluate the presence of overfitting.

In order to reduce bias, five random seed values were utilized for data splitting during data preprocessing [45]. Then, the mean AUC value, the average F1 score, the mean balanced accuracy value, the average precision value, the average recall value, the average FPR value, and the average FNR value were calculated from five repetitions of the data modelling process. The estimates of model performance were obtained through the implementation of 10-stratified k-fold cross-validation in each repetition. This approach ensured the consistent class distribution in each fold, thereby enhancing the reliability of the estimates.

Table S1 listed the best hyperparameter of five traditional ML models for the prediction of EGC in this study. 

### 2.5. The Procedure of Data Modelling of the KAN Method

In this study, an efficient implementation of the KAN architecture, as detailed in [46], was employed. In our experiments, we used a basic KAN model with a single input layer, an intermediate layer configured with ten distinct dimensions of k nodes where k ∈ [6,15], and an output layer for classification [23]. As an activation function, we used the sigmoid linear unit (SiLU) [47]. The detail of the implementation of KANs can be found in the work of Eleonora Poeta et al. [23].

In order to ensure the reliability of the results, five random seed values were utilized for data splitting and then each experiment was conducted five times with different random seeds. When the value of i is an integer within the range [6,15], the intermediate layer of each KAN model is configured with i nodes. In each run, every model is trained for 12 epochs using AdamW [48] optimizer with a learning rate of 10^−2^. Additionally, we apply an exponential decay function to the learning rate, with a decay factor 0.8 [23]. In our study, a comparison was conducted between the metrics of KAN models that had an intermediate layer configured with different numbers of nodes, as calculated in the test set. The results demonstrated that the optimal KAN model is configured with 8 nodes in the intermediate layer in each experiment.

Therefore, seven evaluation metrics were calculated on the testing sets for purpose of assessing the performance of each optimal KAN model in each repetition of the data modelling process. Finally, the average AUC value was calculated from five repetitions of the data modelling process of the KAN model. Other evaluation metrics were obtained in similar way. The code for the KAN is available at https://github.com/eleonorapoeta/benchmarking-KAN (accessed on 19 July 2024).

Table A1 (in the Appendix A) presents the confusion matrix of the KAN model for the prediction of label versus the observed label in a testing set. The false positive rate, false negative rate, positive predictive value, and negative predictive value were calculated according to the confusion matrix. The number of misclassified EGC samples with the Kyoto classification score < 4 and ≥4 and their respective proportions were presented.

### 2.6. The Method for Calculating Feature Importance Score of Variables

In order to assess the association between individual features and the accuracy of a trained model, we also investigated the contribution of each feature on the model training process by calculating the importance score of the predictors included in the model. This score is mainly from the RF model. In RF, the importance score of each feature is calculated by how much each feature improves the error rate of the classifier. The general concept of the impact of a predictor variable in predicting the response is termed “variable importance”. The variable importance measure used in random forests, the Gini importance, is based on the principle of impurity reduction [49]. Finally, the average importance score of all trees in the RF is calculated to obtain the final score for each feature.

## 3. Results

### 3.1. Patient Characteristics

We included 1999 patients who underwent a gastrointestinal endoscopy and routine medical consultations at our institution. Their mean (SD) age was 60.8 (11.1) years, and 1112 patients (55.6%) were men. Among these patients, 203 samples were diagnosed with EGC, confirmed by the endoscopic and histologic findings. The definitions of the variables are shown in Table 2.

### 3.2. Model Performance

The two types of samples included in the testing set are imbalanced, as demonstrated in Table 2. Accordingly, the value of AUC has a higher priority than accuracy for addressing the unbalanced binary classification problem. Consequently, it is identified as the most appropriate metric for evaluating ML models in our study.

The performances of six different ML models for predicting EGC are summarized in Table 3. After repeating the data modelling process five times on a testing set, the performance was evaluated using seven metrics, with the results expressed as mean ± standard deviation. Results showed that the KAN model outperformed other ML models with the highest average AUC value of 0.76, the highest average balanced accuracy of 70.96%, the highest average F1 score value of 58.46, the highest mean precision value of 58.91%, the highest mean recall value of 70.96%, and the lowest false negative rate of 31.96%, respectively. In terms of the metrics of AUC value and F1 score, the KAN model exhibited the lowest standard deviation, indicating a more stable performance in predicting EGC.

The RF model achieved the highest average AUC value of 0.76 and the lowest false positive rate of 25.27%, while the standard deviation of the AUC value of the RF model is slightly larger than the standard deviation of the AUC value of the KAN model.

**Figure A1:**
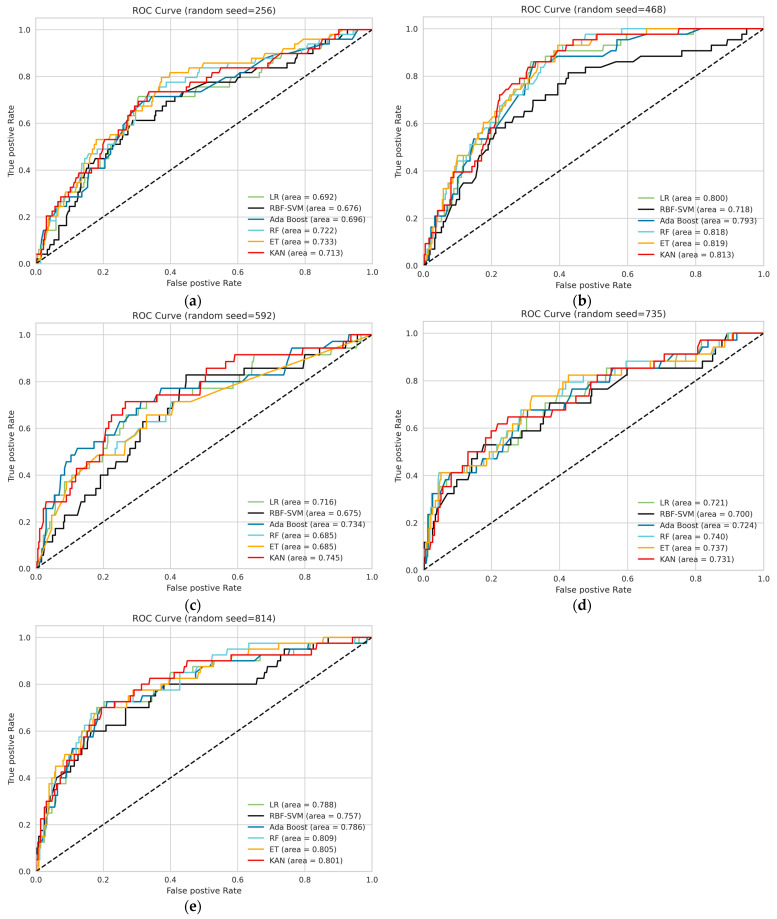
Receiver operating characteristic (ROC) curves and area under curve (AUC) values for six different ML models was obtained by after repeating the data modelling process five times with five different random seed values. (**a**) ROC curves and AUC value in the first repetition of the data modeling process; (**b**) ROC curves and AUC values in the second repetition of the data modeling process; (**c**) ROC curves and AUC value in the third repetition of the data modeling process; (**d**) ROC curves and AUC value in the fourth repetition of the data modeling process; (**e**) ROC curves and AUC value in the fifth repetition of the data modeling process.

Receiver operating characteristic (ROC) curves and area under curve (AUC) values for six different ML models were obtained after repeating the data modelling process five times with five different random seed values (Figure A1 in the Appendix B).

### 3.3. The Result of Feature Importance Score

**Figure 4:**
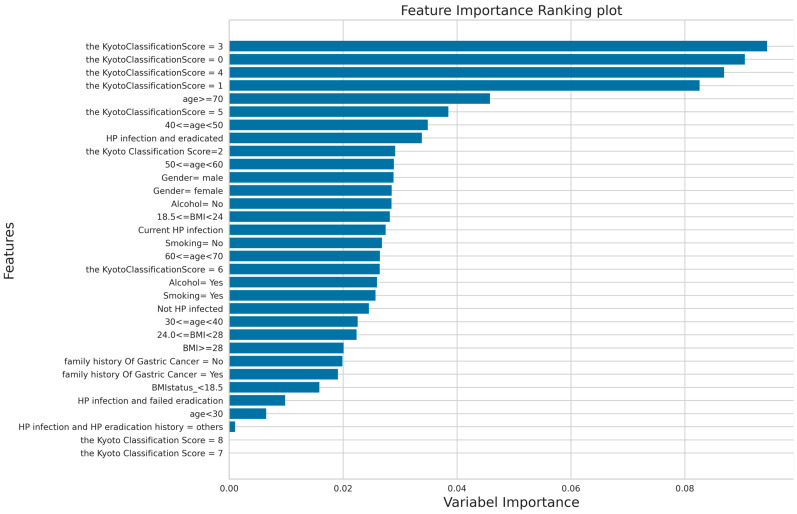
The feature importance score of each variable in the RF model.

In addition to the model performance, we ranked the features in the model training stage according to the feature importance score generated by the RF model, as shown in Figure 4. According to the result of the feature importance score, we conclude that the five variables of the endoscopy-based Kyoto classification score, age, history of HP infection and HP eradication, gender, and alcohol consumption contribute the most. Among these features, the endoscopy-based Kyoto classification score was the most important feature.

## 4. Discussion

The accuracy of early detection of EGC determines the choice of the optimal treatment strategy and the related medical expenses as well as in the prevention of the progression of gastric cancer. Gastrointestinal endoscopy, gradually popularized in primary hospitals, is the primary and preferred means of screening GC. Despite a decline in the incidence of GC and an improvement in people’s living standards and attention to their health over recent decades, the incidence rate of GC in China still accounts for 44% of the world’s gastric cancer incidence rate [1]. Therefore, there is a pressing need to identify a methodology that not only raises the precision of EGC screening but also does so without adding to the financial burden on patients. Thus, there is dire demand to develop a simple, affordable, and time-saving diagnostic model using ML algorithms for the diagnosis of EGC following the completion of gastrointestinal endoscopy and routine consultations. Such a strategy would not only result in the acceptable medical cost, but also contribute to the reduction of the time cost for obtaining a definitive diagnosis. In addition, the required patient’s information is collected from medical consultation, which is readily available in many digestive system departments of medical institutions.

In our study, the diagnostic ability of six ML models for predicting EGC was established based on the endoscopy-based Kyoto classification score and seven traditional risk factors obtained after a simple medical consultation (i.e., age, gender, BMI, family history of gastric cancer, history of HP infection and HP eradication, and smoking and alcohol consumption). The performance of each model was demonstrated according to the mean of the AUC value, balanced accuracy, F1 score, precision, recall, FPR, and FNR metrics, which were obtained after five repetitions of the data modelling process.

Among these ML models, the KAN model has the best performance with the highest average AUC value of 0.76, the highest average balanced accuracy of 70.96%, the highest average F1 score value of 58.46, the highest mean precision value of 58.91%, the highest mean recall value of 70.96%, and the lowest false negative rate of 31.96%, respectively. And the KAN model demonstrated the lowest standard deviation in three metrics of F1 score and AUC value, indicating a more stable performance in predicting EGC. These two points suggest that the KAN model is the optimal ML model for developing a practical and low-cost diagnostic method for EGC using the endoscopy-based Kyoto classification score and multiple clinical features that were easily collected from medical consultation. Previous studies have demonstrated that a Kyoto classification score of 0, ≥2, and ≥4 indicate a normal stomach, *H. pylori*-infected gastritis, and gastritis at risk for GC, respectively [4,50,51]. These findings are consistent with our findings, i.e., the endoscopy-based Kyoto classification score was the most important feature for predicting EGC cases in our study. Our findings, when considered alongside those of previous studies, indicate that the Kyoto classification score plays a critical role in the diagnosis of EGC. This is because it enables the severity of gastritis and the characteristics of visible lesions under endoscopy to be captured, thereby providing a comprehensive assessment tool.

Some researchers use endoscopic features (atrophy, intestinal metaplasia, enlarged fold, nodularity, and diffuse redness), the serum biomarkers of GC (i.e., PG I test, PG II test, and G-17 tests), personal baseline information (age, sex, and BMI), and other information about lifestyle behaviors (diet, drinking, and smoking) to predict the risk of GC. The comparison between the results of other studies and the results of our research is shown in Table 4. For example, Cai Q et al. [13] developed a prescreening tool that comprised the variables age, sex, PG I/II ratio, G-17, anti-*H. pylori* IgG concentrations, and consumption of pickled food and fried food, for identifying individuals at an increased risk of GC in the Chinese high-risk population. The novel GC risk prediction rule showed good performance, with an area under curve of 0.76, which was consistent with the results of our study in terms of the AUC value. Similarly, the research of Lin J et al. [4] demonstrated that the AUC of a predictive nomogram to predict GC using the Kyoto classification score, age, sex, PG I/II ratio, HP antibody, and four endoscopic features was 0.79, which was slightly better than the AUC value results of our study.

However, several differences were observed in the data modelling algorithms and the selected variables in the previous study (Cai Q et al.; Lin J et al.) and those employed in the present study. Firstly, the number of features employed in the present study is fewer. Secondly, our study did not use the serum biomarkers of GC (i.e., PG I test, PG II test, and/or G-17 tests) to train the EGC predictive models, which reduced medical expenses and time cost required to obtain the result of diagnosis. Thirdly, our study constructed EGC prediction models that employed not only five traditional ML algorithms that have been previously applied in previous studies but also using the KAN algorithm. KANs are capable of accurately representing continuous functions while maintaining the interpretability of the underlying model, which may be the reason for its superior performance in our study. The application of KANs can significantly improve the accuracy and interpretability of the function approximation task, which is achieved by representing a function as the sum of these learnable functions [39].

In our study, the mean false negative rate of six ML models evaluated in the testing set ranged from 31.96% to 43.69%. This indicates that the proportion of positive samples (EGC cases) were incorrectly classified as negative (non-EGC cases), resulting in a missed diagnosis. As demonstrated in Table A1, the results of the sample set incorrectly classified by the KAN model indicate that [25.58%, 42.85%] of the EGC samples in the testing set was predicted to be non-EGC samples, and [81.8%, 100%] of misclassified EGC samples with the Kyoto classification score below 4. Previous research may interpret that the main reason for missed diagnosis of EGC cases in the present study. To illustrate, the systematic review and meta-analysis proposed by Zhang H. et al. [6] revealed that intestinal metaplasia and gastric atrophy, both of which influence the score of the Kyoto classification system, were the two factors that had the greatest impact on the risk of GC following the investigation. Moreover, previous research [52] has demonstrated that individuals with intestinal metaplasia and high-level gastric atrophy are at an extremely high risk of developing GC, even after the eradication of *H. pylori*, due to the high incidence and hidden early symptoms of gastric cancer and colon cancer. As demonstrated by the work of Kato M and Kamada T [27], the Kyoto classification score for no-atrophy (C0) and atrophy (C1), mild atrophy (C2–C3), and moderate to severe atrophy (O1–O3) is 0, 1, and 2, respectively. This is the same grading rule as that used in our study. Therefore, we hypothesized that this may be attributable to the presence of intestinal metaplasia and gastric atrophy or the presence of high-level gastric atrophy in these samples, which leads to the development of early gastric cancer in those stomachs.

On the other hand, the mean false positive rate of the six ML models evaluated in the testing set ranged from 25.27% to 27.28%. This resulted in the erroneous classification of some non-EGC cases. In this study, the limited sample size of EGC patients resulted in a difference in the learning degree between positive and negative samples during the training process. This indicates that patients with non-EGC are easy to overfitting, whereas those with EGC are prone to underfitting. This provides an explanation as to why the FNR value of each ML model evaluated in the testing set is higher than its FPR value.

The prevalence rate of gastric cancer is 10.15% (203/1999) in this study; there is a binary classification problem of imbalance in training samples. The resampling process detailed in Section 2.2 was applied to the training set only for the purpose of oversampling positive label samples and under-sampling those with negative labels. This was done in order to balance the learning degree of the two types of labels. The main cause of the very high negative predictive value (see Table A1) is low prevalence of EGC. This made the NPV metric inapplicable to the detection of rare diseases, as it failed to provide sufficient information to assess the performance of the model [53].

Other features, such as age, gender, BMI, and *H. pylori* infection, are associated with early gastric cancer. Each of these features has a specific role to play in screening, and they cannot be substituted for one another. Similarly, the Kyoto classification score cannot be replaced by other features. In future research, we intend to investigate how these features interact and complement each other in the risk assessment framework.

There are some limitations in this study: First, our study was a single-center retrospective analysis, which may introduce bias into dataset. However, our research is particularly significant due to the unique patient population we have access to at the National Center of Gerontology, which serves as the national referral center for geriatric health issues. Our patient pool is not limited to local residents but encompasses individuals from all over the country. We acknowledge the importance of sample diversity and the limitations of sampling bias. In future research, we plan to develop multicenter studies to minimize the impact of sampling bias on the results. Second, the total size of the sample was relatively small, with only 203 EGC cases enrolled. However, the low prevalence rate of EGC makes it challenging to collect sufficient EGC cases. Nevertheless, the resampling process of training sets during data preprocessing ensures that the predictive model for EGC remains valid despite the imbalanced training datasets in our study. In future research, we will increase the sample size to enhance statistical efficacy. Third, intestinal metaplasia and gastric atrophy, the two endoscopic findings that definitively effect the development of EGC, could not concretely describe in the endoscopy-based Kyoto classification score separately. Future studies should incorporate clinical features related to intestinal metaplasia and gastric atrophy in order to develop new EGC diagnostic models with the aim of improving the predictive performance of EGC. Fourth, the Kyoto classification score has its own limitations in the assessment process, like depending on the seniority and experience of the endoscopist, and overcoming this was also the original intention of our study. We aim to compile the subjective and objective factors related to gastric cancer screening, which are readily available in primary care hospitals, into a computer model that is both accurate and convenient for primary care physicians. Lastly, we employed ML methods and tabular data for constructing EGC prediction model instead of deep learning methods. However, previous research reported that the uses of transfer learning in the mutation detection of different cancers (lung, gastrointestinal, breast, and glioma), gene expression, and genetic syndrome detection based on the phenotype of patients [54]. Using transfer learning in model development improves the final performance of the model compared with models trained from scratch [54]. Therefore, we intend to investigate the potential of different types of deep learning methods and other complex data types, such as endoscopic images and serological tests, for the diagnosis of EGC in future research.

## 5. Conclusions

The present study demonstrates that using ML algorithms based on the endoscopy-based Kyoto classification score and other clinical features collected from medical consultations is an effective method to identify EGC patients in China. The endoscopic Kyoto classification of gastritis, obtained following the completion of endoscopy, has the potential to significantly enhance the precision of EGC prediction models. Introducing the optimal ML model into clinical practice, such as the KAN model, maybe be beneficial to reducing the risk of missed diagnoses of EGC following gastrointestinal endoscopy and routine consultations, and medical expenses, as well as saving time for receiving a definitive diagnosis.

## Figures and Tables

**Table 1:** The number of positive samples and negative samples in a training set and a testing set after data preprocessing.

The Binary Classification	The Serial Number of 5 Repetitions When Using 5 Different Random Seed Values for Data Splitting				
	1	2	3	4	5
	Random Seed = 256	Random Seed = 468	Random Seed = 592	Random Seed = 735	Random Seed = 814
Number of samples with the positive class label in a training set after data preprocessing	841	775	790	809	793
Number of samples with the negative class label in a training set after data preprocessing	758	824	809	790	806
Number of samples with the positive class label in a testing set after data preprocessing	49	43	35	34	40
Number of samples with the negative class label in a testing set after data preprocessing	351	357	365	366	360

**Table 2:** The descriptive statistics of variables.

Variable Name	Attribute and Type	Values	With Early Gastric Cancer (203)	No Early Gastric Cancer (1796)	Total
Age (years)	Categorical variable	<30	1	14	15
		30–39	0	64	64
		40–49	8	219	227
		50–59	35	510	545
		60–69	82	626	708
		≥70	77	363	440
BMI	Categorical variable	<18.5	15	106	121
		18.5–23.9	88	796	884
		24–27.9	78	729	807
		≥28	22	165	187
Gender	Categorical variable	Male	132	980	1112
		Female	71	816	887
HP infection and HP eradication history	Categorical variable	Previous infection and eradicated	90	668	758
		Previous infection and failed eradication	5	53	58
		Current infection	27	200	227
		Not HP infected	79	857	936
		Others	2	18	20
Smoking	Categorical variable	No	132	1324	1456
		Yes	71	472	543
Alcohol	Categorical variable	No	138	1289	1427
		Yes	65	507	572
Family history of gastric cancer	Categorical variable	No	176	1612	1788
		Yes	27	184	211
The endoscopy-based Kyoto classification score	Categorical variable	0	23	593	616
		1	25	567	592
		2	39	329	368
		3	49	177	226
		4	39	68	107
		5	18	48	66
		6	9	13	22
		7	1	0	1
		8	0	1	1

**Table 3:** The means and standard deviations of performance metrics of six machine learning models for the prediction of EGC using all features (the highest average F1 score and average AUC value is in bold).

Metrics	ET	Ada Boost	LR	RF	RBF-SVM	KAN
AUC	0.758 ± 0.05	0.744 ± 0.05	0.742 ± 0.05	0.760 ± 0.05	0.691 ± 0.05	**0.760 ± 0.04**
F1 score	57.87 ± 3.34	57.23 ± 3.95	58.07 ± 2.99	57.68 ± 3.82	55.05 ± 4.89	**58.46 ± 2.73**
Precision	58.29 ± 2.15	58.03 ± 2.13	58.45 ± 1.85	58.11 ± 2.59	56.33 ± 3.47	58.91 ± 1.91
Recall	68.84 ± 3.64	68.49 ± 3.53	69.35 ± 3.25	68.44 ± 5.02	64.52 ± 7.66	70.96 ± 3.99
FPR	25.32 ± 2.56	26.56 ± 4.83	25.38 ± 2.57	25.27 ± 2.75	27.28 ± 2.16	26.11 ± 1.47
FNR	37.01 ± 6.45	36.46 ± 3.59	35.90 ± 5.16	37.86 ± 8.85	43.69 ± 13.45	31.96 ± 7.10
BA	68.83 ± 3.64	68.48 ± 3.53	69.35 ± 3.25	68.43 ± 5.02	64.51 ± 7.66	70.96 ± 3.99

AUC: area under the receiver operating characteristic curve; FPR: false positive rate; FNR: false negative rate; BA: balanced accuracy.

**Table 4:** Comparing machine learning methods to predict EGC patients based on clinical features.

Studies	No. of Patients Enrolled	Characteristics Used for Prediction	No. of Features Collected	Methodology	Result
Cai Q et al. [13] (2019)	14929	Age, sex, BMI, *H. pylori* infection, PG I, PG II, PG I/II ratio, G-17, anti-*H. pylori* IgG antibody, pickled food, fried food, high-salt diet, alcohol consumption and smoking consumption, etc.	21	Logistic regression	The prediction rule owns a good discrimination, with an AUC of 0.76.
Lin J et al. [4] (2022)	2639	Age, sex, PG I/II ratio, HP antibody, atrophy, intestinal metaplasia, enlarged fold, diffuse redness and the Kyoto classification score	9	Nomogram	The AUC of the nomogram to predict GC was 0.79
Our study (2024)	1999	The Kyoto classification score, age, gender, BMI, family history of gastric cancer, the history of *H. Pylori* infection and *H. Pylori* eradication, smoking consumption and alcohol consumption	8	Logistic regression, extra trees, radial basis function kernel support vector machine, Ada Boost, random forest, Kolmogorov–Arnold networks	The KAN model outperformed other ML models with the highest average AUC value of 0.76, the highest average balanced accuracy of 70.96%.

G-17: gastrin-17; *H. pylori*: *Helicobacter pylori*; PG: pepsinogen; AUC: area under the receiver operating characteristic curve.

## Data Availability

The data presented in this study are available on request from the corresponding author.

## Supplementary Materials

The following supporting information can be downloaded at: https://www.mdpi.com/article/10.3390/bioengineering11100973/s1, Table S1: The list of the best hyperparameter of five traditional ML models for the prediction of EGC in this study.

## Appendix A

**Table A1:** The confusion matrix of the KAN model for the prediction of label versus the observed label in a testing set. The false positive rate, false negative rate, positive predictive value, and negative predictive value were calculated according to the confusion matrix. The number of misclassified EGC samples with the Kyoto classification score < 4 and ≥4 and their respective proportions were presented.

The Binary Classification	The Serial Number of 5 Repetitions When Using 5 Different Random Seed Values for Data Splitting				
	1	2	3	4	5
	Random Seed = 256	Random Seed = 468	Random Seed = 592	Random Seed = 735	Random Seed = 814
True negative (TN)	257	270	264	267	271
False positive (FP)	94	87	101	99	89
False negative (FN)	21	11	10	12	11
True positive (TP)	28	32	25	22	29
Number of misclassified EGC samples with the Kyoto classification score < 4	18	9	10	11	9
Number of misclassified EGC samples with the Kyoto classification score ≥ 4	3	2	0	1	2
The ratio of misclassified EGC samples with the Kyoto classification score < 4	85.7%	81.8%	100%	91.7%	81.8%
The ratio of misclassified EGC samples with the Kyoto classification score ≥ 4	14.3%	18.2%	0%	8.3%	18.2%
False positive rate (FPR)	26.78%	24.36%	27.67%	27.04%	24.72%
False negative rate (FNR)	42.85%	25.58%	28.57%	35.29%	27.50%
Positive predictive value (PPV)	22.95%	26.89%	19.84%	18.18%	24.57%
Negative predictive value (NPV)	92.44%	96.08%	96.35%	95.69%	96.09%

Here, the true positive (TP) value refers to the number of positive class instances that are predicted by the model correctly; the true negative (TN) value refers to the number of negative class instances that are predicted by the model correctly; the false positive (FP) value refers to the number of negative instances that are incorrectly predicted as positive by the model; the false negative (FN) value refers to the number of positive instances that are incorrectly predicted as negative by the model.
