# Supplementary material for: Application of Machine Learning in the Diagnosis of Early Gastric Cancer Using the Kyoto Classification Score and Clinical Features Collected from Medical Consultations
Source: Bioengineering (Basel). 2024 Sep 27;11(10):973. doi: 10.3390/bioengineering11100973 (PMC11504958; doi:10.3390/bioengineering11100973)
Supplement: Supplementary file 1 [file bioengineering-11-00973-s001.zip › bioengineering-3168514-supplementary.pdf]

Table S1. The list of the best hyper-parameter of five traditional ML models for the prediction of EGC in this study.

Name of Five ML methods	the serial number of 5 repetition when using 5 different random seed value for data splitting				
	1	2	3	4	5
	random seed=256	random seed=468	random seed=592	random seed=735	random seed=814
Logistic Regression Classifier (LR)	(C=8.51, class_weight='balanced', fit_intercept=True, intercept_scaling=1, l1_ratio=None, max_iter=1000, multi_class='auto', penalty='l2', random_state=256, solver='lbfgs', tol=0.0001)	(C=6.588, class_weight='balanced',fit_intercept=True, intercept_scaling=1, l1_ratio=None, max_iter=1000, multi_class='auto', penalty='l2', random_state=468, solver='lbfgs', tol=0.0001)	(C=4.821, class_weight='balanced',fit_intercept=True, intercept_scaling=1, l1_ratio=None, max_iter=1000, multi_class='auto', penalty='l2', random_state=592, solver='lbfgs', tol=0.0001)	(C=8.904, class_weight='balanced',fit_intercept=True, intercept_scaling=1, l1_ratio=None, max_iter=1000, multi_class='auto', penalty='l2', random_state=735, solver='lbfgs', tol=0.0001)	(C=4.962, class_weight='balanced', fit_intercept=True, intercept_scaling=1, l1_ratio=None, max_iter=1000, multi_class='auto', penalty='l2', random_state=814, solver='lbfgs', tol=0.0001)
Radial Basis Function Kernel Support Vector Machine Classifier (RBF-SVM)	(C=47.61, coef0=0.0, decision_function_shape='ovr', degree=3, gamma='auto', kernel='rbf', max_iter=-1, probability=True, random_state=256, shrinking=True, tol=0.001)	(C=49.13, coef0=0.0, decision_function_shape='ovr', degree=3, gamma='auto', kernel='rbf', max_iter=-1, probability=True, random_state=468, shrinking=True, tol=0.001)	(C=48.2, coef0=0.0, decision_function_shape='ovr', degree=3, gamma='auto', kernel='rbf', max_iter=-1, probability=True, random_state=592, shrinking=True, tol=0.001)	(C=44.36, coef0=0.0, decision_function_shape='ovr', degree=3, gamma='auto', kernel='rbf', max_iter=-1, probability=True, random_state=735, shrinking=True, tol=0.001)	(C=49.61 coef0=0.0, decision_function_shape='ovr', degree=3, gamma='auto', kernel='rbf', max_iter=-1, probability=True, random_state=814, shrinking=True, tol=0.001)
Random Forest Classifier (RF)	(class_weight='balanced_subsample', criterion='entropy', max_depth=11, max_features='log2', min_impurity_decrease=0, min_samples_leaf=6, min_samples_split=7, min_weight_fraction_leaf=0.0, n_estimators=110, random_state=256)	(class_weight='balanced', criterion='entropy', max_depth=8, max_features='log2', min_impurity_decrease=0.0002, min_samples_leaf=3, min_samples_split=2, min_weight_fraction_leaf=0.0, n_estimators=250, random_state=468)	(class_weight='balanced', criterion='entropy', max_depth=9, max_features='log2', min_impurity_decrease=0.0002, min_samples_leaf=4, min_samples_split=10, min_weight_fraction_leaf=0.0, n_estimators=300, random_state=592)	(class_weight='balanced_subsample', criterion='gini', max_depth=5, max_features='sqrt', min_impurity_decrease=0.0002, min_samples_leaf=3, min_samples_split=7, min_weight_fraction_leaf=0.0, n_estimators=220,random_state=735)	(class_weight='balanced_subsample', criterion='entropy', max_depth=8, max_features='log2', min_impurity_decrease=0.0002, min_samples_leaf=4, min_samples_split=10, min_weight_fraction_leaf=0.0, n_estimators=100, random_state=814)

Extra Trees Classifier (ET)	(class_weight='balanced_subsample', criterion='entropy', max_depth=11, max_features='log2', min_impurity_decrease=0, min_samples_leaf=6, min_samples_split=7, min_weight_fraction_leaf=0.0, n_estimators=110, random_state=256)	(class_weight='balanced', criterion='entropy', max_depth=8, max_features='log2', min_impurity_decrease=0.0002, min_samples_leaf=3, min_samples_split=2, min_weight_fraction_leaf=0.0, n_estimators=250, random_state=468)	(class_weight='balanced', criterion='entropy', max_depth=9, max_features='log2', min_impurity_decrease=0, min_samples_leaf=4, min_samples_split=10, min_weight_fraction_leaf=0.0, n_estimators=300, random_state=592)	(class_weight='balanced_subsample', criterion='entropy', max_depth= 5, max_features= 'sqrt', min_impurity_decrease=0.0002, min_samples_leaf=3, min_samples_split=7,min_weight_fraction_leaf=0.0, n_estimators=220, random_state=735)	(class_weight='balanced_subsample', criterion='entropy', max_depth=8, max_features='log2', min_impurity_decrease=0.0002, min_samples_leaf=4, min_samples_split=10, min_weight_fraction_leaf=0.0, n_estimators=100, random_state=814)
Ada Boost Classifier (Ada Boost)	(algorithm= 'SAMME.R', base_estimator=None, learning_rate=0.15, n_estimators=90, random_state=256)	(algorithm= 'SAMME.R', base_estimator=None, learning_rate=0.3, n_estimators=280, random_state=468)	(algorithm= 'SAMME.R', base_estimator=None, learning_rate=0.15, n_estimators=90, random_state=592)	(algorithm= 'SAMME.R', base_estimator=None, learning_rate=0.1, n_estimators=290, random_state=735)	(algorithm= 'SAMME.R', base_estimator=None, learning_rate=0.3, n_estimators=140, random_state=814)
